# Intravascular ultrasound-guided tip detection-antegrade dissection re-entry as a bailout technique for coronary artery dissection: a case report

**DOI:** 10.1093/ehjcr/ytag162

**Published:** 2026-03-06

**Authors:** Shintaro Izumoto, Kazumasa Kurogi, Yunosuke Matsuura, Nobuyasu Yamamoto, Koichi Kaikita

**Affiliations:** Division of Cardiovascular Medicine and Nephrology, Department of Internal Medicine, Faculty of Medicine, University of Miyazaki, Miyazaki, Japan; Department of Cardiovascular Medicine, Miyazaki Prefectural Nobeoka Hospital, Miyazaki, Japan; Department of Cardiovascular Medicine, Miyazaki Prefectural Nobeoka Hospital, Miyazaki, Japan; Division of Cardiovascular Medicine and Nephrology, Department of Internal Medicine, Faculty of Medicine, University of Miyazaki, Miyazaki, Japan; Department of Cardiovascular Medicine, Miyazaki Prefectural Nobeoka Hospital, Miyazaki, Japan; Division of Cardiovascular Medicine and Nephrology, Department of Internal Medicine, Faculty of Medicine, University of Miyazaki, Miyazaki, Japan

**Keywords:** Percutaneous coronary intervention, Coronary artery dissection, Intravascular ultrasound, Tip detection-antegrade dissection re-entry, Bailout technique, Case report

## Abstract

**Background:**

Coronary artery dissection during percutaneous coronary intervention (PCI) may lead to haemodynamic collapse. Intravascular ultrasound (IVUS)-guided tip detection-antegrade dissection re-entry (TD-ADR) may serve as a bailout strategy. We describe a unique case in which IVUS-guided TD-ADR successfully achieved true lumen re-entry after severe coronary artery dissection.

**Case summary:**

A 64-year-old woman who had received long-term immunosuppressive therapy for rheumatoid arthritis was admitted for unstable angina. Subsequent evaluation revealed severe multivessel disease, and the patient underwent PCI of the left anterior descending artery (LAD). During the procedure, the patient experienced cardiac arrest, likely due to worsening global myocardial ischaemia, and required venoarterial extracorporeal membrane oxygenation support. IVUS demonstrated subintimal wire tracking with true lumen collapse. IVUS-guided TD-ADR using a high-penetration chronic total occlusion wire supported by a microcatheter enabled precise re-entry into the true lumen and successful LAD revascularization. Severe left circumflex artery stenoses were also considered to perpetuate myocardial ischaemia and haemodynamic instability, necessitating rescue PCI for haemodynamic stabilization. In addition, major bleeding complications due to guidewire-induced perforation of the right subclavian artery branch were managed using stenting and coil embolization. The patient showed recovery of left ventricular function and was discharged in stable condition. During the 8-month follow-up after discharge, the patient was free of adverse events.

**Discussion:**

This case highlights the feasibility of IVUS-guided TD-ADR as a bailout technique for coronary artery dissection during complex PCI. By enabling accurate true lumen re-entry, this approach facilitates timely revascularization and contributes to haemodynamic stabilization and recovery in a life-threatening setting.

Learning points

- To highlight the role of IVUS-guided TD-ADR as a precise true lumen re-entry technique and an effective bailout strategy during high-risk PCI complicated by coronary artery dissection.
- To demonstrate the technical feasibility of performing TD-ADR with conventional IVUS catheters.

## Introduction

Patients with severe multivessel disease undergoing percutaneous coronary intervention (PCI) are at high risk of acute haemodynamic deterioration. Mechanical circulatory support devices can provide temporary stabilization, but prognosis remains poor without timely and successful revascularization of the culprit vessel. Complex PCI is associated with technical challenges and serious complications, among which guidewire-induced coronary artery dissection is particularly critical. However, reproducible bailout strategies for this complication remain limited.

Intravascular ultrasound (IVUS)-guided tip detection-antegrade dissection re-entry (TD-ADR) has been reported as an effective revascularization technique for chronic total occlusion (CTO) lesions^1,2^ and ST-segment elevation myocardial infarction (STEMI)^3^ when conventional wiring fails. This method allows precise identification of re-entry sites under real-time IVUS guidance; however, its role as a bailout strategy for coronary artery dissection has been insufficiently explored. Here, we describe a case in which IVUS-guided TD-ADR successfully achieved true lumen re-entry after severe coronary artery dissection with intramural haematoma and true lumen collapse, thereby enabling life-saving revascularization.

## Summary figure

**5 months before admission:** Intermittent chest pain, progressively worsening

**Day 0:** Referred with unstable angina

**Day 1:**

**Initial deterioration and PCI**

- Recurrent chest pain with ECG changes indicating global myocardial ischaemia after admission.
- Emergent coronary angiography revealed severe multivessel disease; urgent PCI to the LAD was performed.

**Cardiac arrest and mechanical circulatory support**

- Multiple guidewires in the LAD and its side branch limited coronary flow specifically at the critically stenotic LAD segment, precipitating cardiac arrest.
- VA-ECMO was initiated following cardiac arrest.

**Bailout revascularization**

- During resuscitation, guidewires were dislodged; the LAD was re-crossed using a polymer-jacketed guidewire.
- IVUS demonstrated subintimal wire tracking with true lumen collapse; IVUS-guided TD-ADR was then performed.
- Successful LAD revascularization was achieved, along with additional PCI to other lesions.

**Post-PCI haemodynamic management and complications**

- Impella CP was inserted for persistently elevated pulmonary capillary wedge pressure.
- Impella was removed because of guidewire-induced bleeding from a right subclavian artery branch, which was treated with covered stenting and coil embolization.
- Intra-aortic balloon pump was inserted.

**Day 5**: VA-ECMO was removed.

**Day 7**: Intra-aortic balloon pump was removed.

**Day 9**: Left ventricular ejection fraction improved from 16% to 49%.

**Day 12**: ICU care was completed; however, the patient required rehabilitation because of severe deconditioning.

**Day 45**: Discharged in stable clinical condition.

**At 8 months follow-up:** No adverse cardiovascular or cerebrovascular events.

## Case presentation

A 64-year-old woman experienced intermittent chest pain for 5 months, which had recently worsened, prompting consultation at a local clinic. An exercise electrocardiogram (ECG) demonstrated ST-segment depression in widespread leads, accompanied by chest pain. The patient was referred to our hospital for further evaluation. The medical history included dyslipidaemia, rheumatoid arthritis, and hypothyroidism; ongoing treatment included aspirin, bisoprolol fumarate, nicorandil, methotrexate, and folic acid.

**Figure 1:**
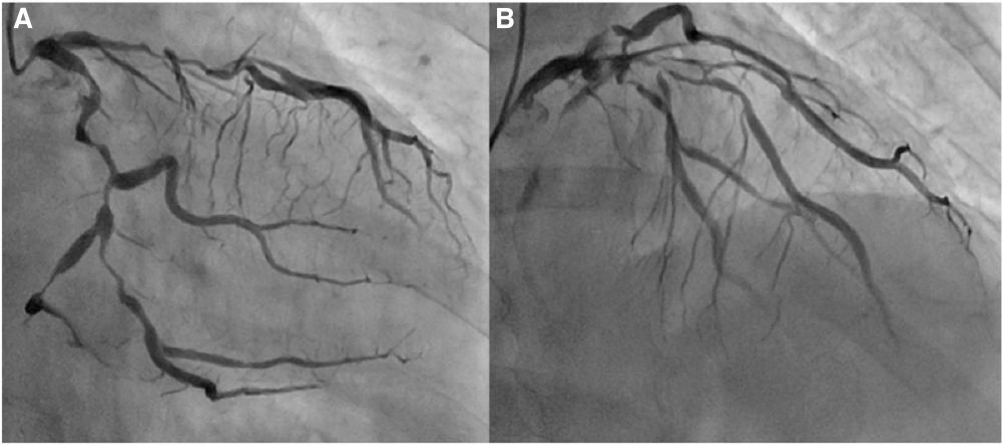
Coronary angiography of the left coronary artery: (*A*) right anterior oblique caudal view and (*B*) straight cranial view.

On admission, her blood pressure was 117/88 mmHg, pulse rate was 67 beats/min, and physical examination revealed normal heart and lung sounds. Transthoracic echocardiography revealed subtle regional wall motion abnormalities in the inferoseptal wall, with preserved left ventricular ejection fraction (63%) and no significant valvular disease. The results of initial laboratory tests, including high-sensitivity troponin T, creatine kinase (CK), and CK-MB, were within normal limits. Shortly after admission, the patient developed recurrent and persistent chest pain despite receiving optimal medical therapy. ECG then revealed persistent widespread ST depression and ST elevation in the aVR, suggesting global myocardial ischaemia. Emergency coronary angiography revealed severe multivessel disease (*Figure 1*).

Despite the patient’s progressive myocardial ischaemia, emergent surgical revascularization was temporarily unavailable at our institution owing to a transient staffing shortage in the cardiovascular surgery team. In addition, long-distance transfer to another surgical centre was considered to carry substantial risk, and the patient’s chronic immunosuppression further increased surgical risk. Therefore, after on-table discussion, the heart team reached a consensus to proceed with PCI.

Following a 20 mg loading dose of prasugrel,^4^ PCI was initiated via the right radial artery using a 7-French guiding catheter (EBU3.5, Medtronic Cardiovascular). A conventional guidewire (SION, Asahi Intecc) was successfully crossed through the left anterior descending artery (LAD) lesion, followed by an attempt to advance the polymer-jacketed guidewire (SION black, Asahi Intecc) into the diagonal branch for side branch protection. During this process, multiple guidewires traversed the critically stenotic LAD lesion, resulting in compromised antegrade coronary flow. Coronary angiography immediately prior to cardiac arrest demonstrated significant flow impairment in the LAD (TIMI grade 2). The patient subsequently developed cardiac arrest, likely due to worsening global myocardial ischaemia, and required venoarterial extracorporeal membrane oxygenation support.

During resuscitation, the guidewire slipped out of the LAD, and re-crossing the lesion proved difficult. IVUS revealed subintimal guidewire tracking with collapse of the true lumen in the LAD.

**Figure 2:**
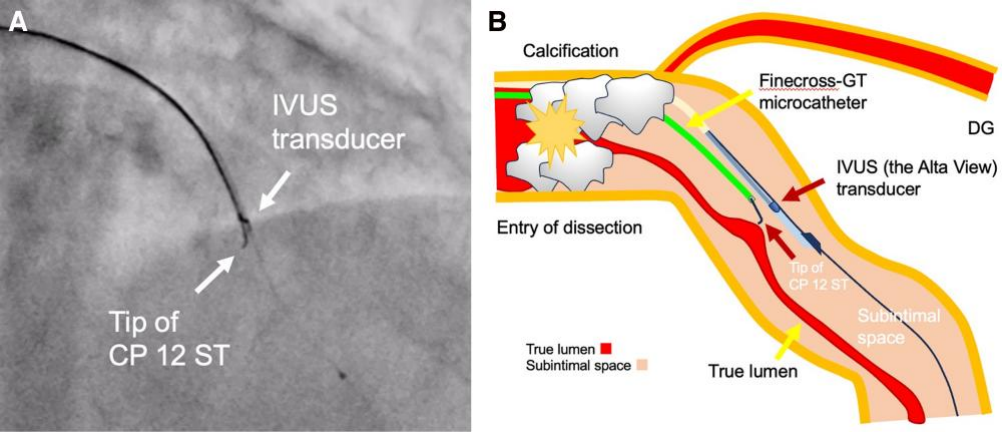
Longitudinal illustration of the IVUS-guided TD-ADR technique. (*A*) Fluoroscopy and (*B*) schematic longitudinal illustration of intravascular ultrasound (IVUS)-guided tip detection-antegrade dissection re-entry (TD-ADR). CP: Conquest Pro; DG: diagonal branch; IVUS: intravascular ultrasound; TD-ADR: tip detection-­antegrade dissection re-entry.

**Figure 3:**
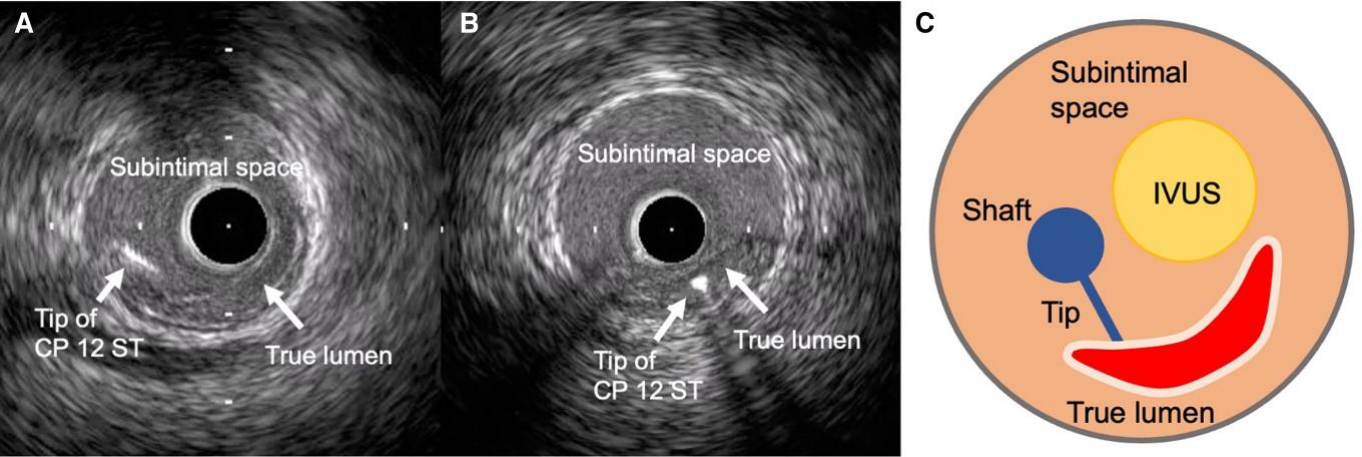
Cross-sectional illustration of the IVUS-guided TD-ADR technique. **(***A, B***)** IVUS images during TD-ADR and (*C*) schematic cross-sectional illustration of IVUS-guided TD-ADR. CP: Conquest Pro; IVUS: intravascular ultrasound; TD-ADR: tip detection-antegrade dissection re-entry.

**Figure 4:**
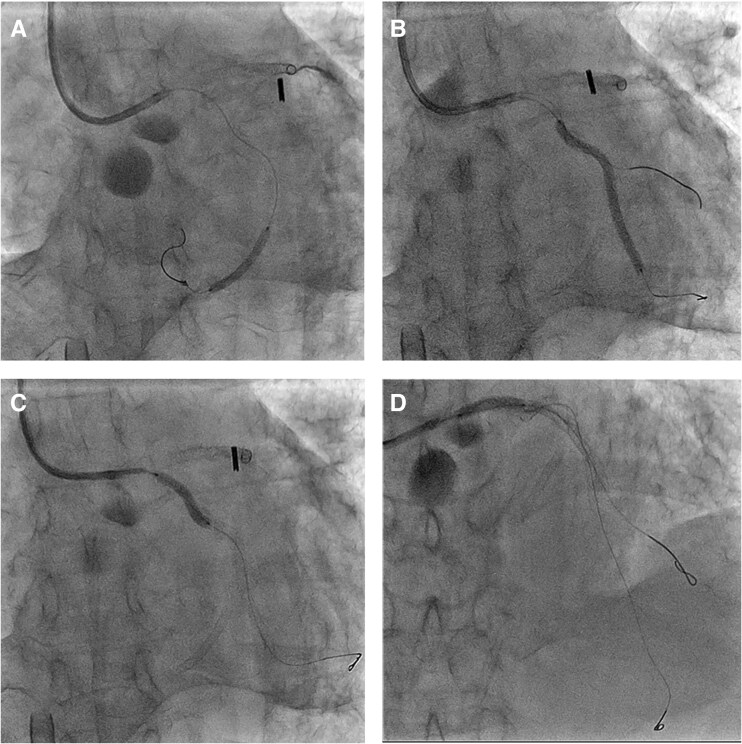
Fluoroscopic images of DESs implantation in the LCX and the LMT to the LAD. (*A*) DES implantation in the LCX segment 15. (*B, C*) DESs implantation in the LCX segments 11–14. (*D*) DES implantation in the LMT to the LAD. DES: drug-eluting stent; LCX: left circumflex coronary artery; LMT: left main trunk; LAD: left anterior descending artery.

**Figure 5:**
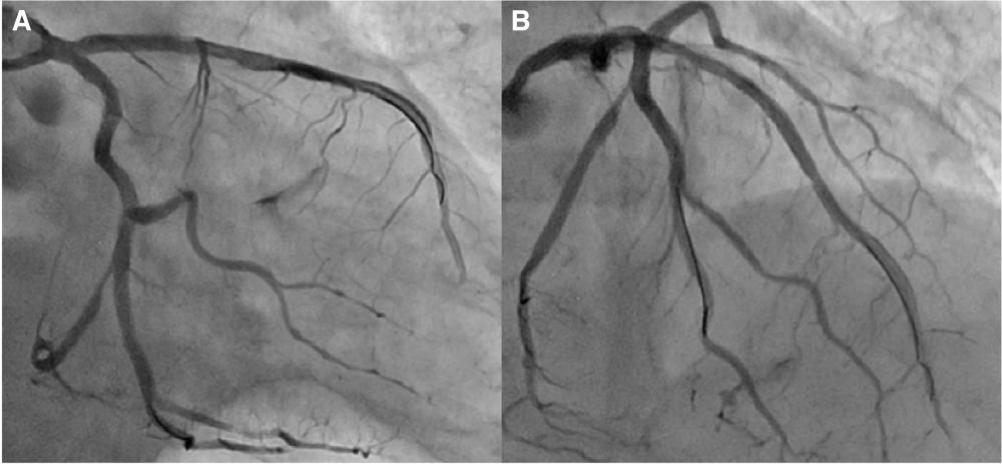
Final coronary angiography after successful revascularization: (*A*) right anterior oblique caudal view and (*B*) straight cranial view.

IVUS-guided TD-ADR was performed using a Conquest Pro 12 ST (CP12ST) wire (Asahi Intecc), supported by a Finecross-GT microcatheter (Terumo Corp). This technique enabled accurate wire orientation and the successful puncture of the collapsed true lumen (*Figures 2* and *3*). Subsequently, a drug-eluting stent (DES) was implanted, successfully restoring LAD flow. Also, severe left circumflex artery (LCX) stenoses were considered to perpetuate myocardial ischaemia and haemodynamic instability; thus, rescue PCI was performed for haemodynamic stabilization, with implantation of a DES in LCX segment 15 and two additional DESs in segments 11 to 14 (*Figure 4A–C*). Post-PCI IVUS demonstrated a dissection extending from the LAD to the left main trunk, which required the implantation of a DES from the left main trunk to the LAD (*Figure 4D*). The final angiography confirmed optimal stent expansion and satisfactory coronary flow (*Figure 5*).

Haemodynamic evaluation using a Swan–Ganz catheter revealed elevated pulmonary artery wedge pressure, prompting the use of Impella CP (Abiomed). Shortly after running the Impella CP, unexpected swelling of the right chest was noted. Contrast-enhanced computed tomography and angiography revealed active bleeding from a branch of the right subclavian artery due to guidewire-induced perforation, which was managed with transfusion, covered stent implantation, and coil embolization, successfully achieving haemostasis. Due to the progression of anaemia and thrombocytopenia, the Impella CP was removed and replaced with an intra-aortic balloon pump. Peak CK and CK-MB levels reached 2584 and 187.5 IU/L, respectively, reflecting side-branch occlusion. Following PCI, transient left ventricular systolic dysfunction was noted with an ejection fraction of 16%. Comprehensive management, including guideline-directed medical therapy, improved the ejection fraction to 49% by day 9. Venoarterial extracorporeal membrane oxygenation was discontinued on day 5, and the intra-aortic balloon pump was removed on day 7. ICU admission was completed on day 12, and the patient was discharged on day 45 in a stable clinical condition. At the 8-month follow-up, the patient remained free of adverse cardiovascular or cerebrovascular events.

## Discussion

Complications and acute haemodynamic compromise can occur during complex PCI in patients with severe multivessel coronary artery disease. Major coronary artery dissection constitutes an infrequent complication of PCI, with an incidence of approximately 1.4%. Despite its rarity, this complication is a potent determinant of poor prognosis, strongly correlating with major adverse cardiovascular events in roughly 23% of affected patients.^5^ In such circumstances, bailout techniques are decisive factors that influence the prognosis.

IVUS-guided TD-ADR has been reported to be effective for revascularization of CTO lesions^1,2^ and STEMI^3^ when conventional wiring fails. This technique enables precise puncture under real-time IVUS guidance, thereby improving the likelihood of successful recanalization. In this case, IVUS played a central role in confirming the subintimal guidewire position and guiding re-entry. Subintimal tracking was identified by the visualization of the intimal flap and the compression of the true lumen. A second operator manipulated the IVUS transducer to align with the guidewire tip, allowing the operator to puncture the partition between the subintima and the true lumen at the intended site in a perpendicular direction. Until recently, ADR in CTO-PCI was widely performed using the Stingray system.^6^ Stingray ADR requires substantial experience to master and has limited accuracy, whereas TD-ADR offers high accuracy but requires a second operator skilled in IVUS and the use of a dedicated guidewire, the CP12ST. In this case, Stingray ADR was considered highly challenging because it depends on angiographic guidance, which was not feasible due to the absence of collateral flow in the ACS setting. In contrast, IVUS-guided TD-ADR is applicable even in ACS because it does not require angiographic visualization of the distal true lumen. The present case illustrates that TD-ADR can also serve as an effective bailout option after coronary artery dissection. Successful revascularization of the LAD through TD-ADR facilitated stabilization and subsequent recovery despite the challenging conditions of cardiac arrest and global myocardial ischaemia.

Although the TD-ADR was originally pioneered using the AnteOwl WR IVUS catheter (Terumo Corp), the limited global availability of this specialized device has restricted its broader adoption. Our case illustrates that TD-ADR can be performed successfully using conventional IVUS equipment, which provides sufficient distal vessel space. Previous reports have described successful TD-ADR using conventional IVUS during PCI for CTO.^7,8^ Our case further supports its feasibility in situations where AnteOwl is unavailable, thereby expanding the applicability of this revascularization strategy.

Another key consideration is that the decision to pursue PCI over surgery was influenced by chronic immunosuppressive therapy. However, a major haemorrhagic complication that required endovascular haemostasis occurred. Therefore, this case illustrates that in vulnerable patients such as those receiving long-term immunosuppressive therapy, clinicians should remain vigilant regarding the occurrence of diverse complications during high-risk PCI and prepare appropriate management strategies.

PCI for complex lesions in vulnerable patients carries a high risk of complications, highlighting the need for meticulous planning of multiple bailout strategies and prompt procedural adaptability. In this setting, IVUS-guided TD-ADR should be recognized as a valuable bailout option.

## Data Availability

All data in this manuscript can be provided upon reasonable request from the corresponding author.

## Lead author biography

Shintaro Izumoto was born in 1991 in Miyazaki, Japan. He graduated from the Faculty of Medicine, University of Miyazaki, in 2016. After completing his residency, he worked at the Emergency and Critical Care Center of the University of Miyazaki, where he became board-certified in emergency medicine. He subsequently joined the Division of Cardiovascular Medicine and Nephrology, Department of Internal Medicine, Faculty of Medicine, University of Miyazaki. He is currently a staff cardiologist at Miyazaki Prefectural Nobeoka Hospital. His clinical interests include cardiovascular emergency medicine and catheter-based interventions.

## Notes

**Consent:** Written informed consent was obtained according to the Committee on Publication Ethics (COPE) guidelines.
